# Simulation-Based and Experimental Investigation of Micro End Mills with Wiper Geometry

**DOI:** 10.3390/mi12050496

**Published:** 2021-04-27

**Authors:** Timo Platt, Alexander Meijer, Torben Merhofe, Dirk Biermann

**Affiliations:** Institute of Machining Technology (ISF), TU Dortmund University, D-44227 Dortmund, Germany; alexander.meijer@tu-dortmund.de (A.M.); torben.merhofe@tu-dortmund.de (T.M.); dirk.biermann@tu-dortmund.de (D.B.)

**Keywords:** micromilling, wiper, material removal simulation, surface roughness, cutting force, AISI H11

## Abstract

One of the major advantages of micromachining is the high achievable surface quality at highly flexible capabilities in terms of the machining of workpieces with complex geometric properties. Unfortunately, finishing operations often result in extensive process times due to the dependency of the resulting surface topography on the cutting parameter, e.g., the feed per tooth, *f*_z_. To overcome this dependency, special tool shapes, called wipers, have proven themselves in the field of turning. This paper presents the transfer of such tool shapes to solid carbide milling tools for micromachining. In this context, a material removal simulation (MRS) was used to investigate promising wiper geometries for micro end mills (*d* = 1 mm). Through experimental validation of the results, the surface topography, the resulting process forces, and tendencies in the residual stress state were investigated, machining the hot work tool steel (AISI H11). The surface-related results show a high agreement and thus the potential of MRS for tool development. Deviations from the experimental data for large wipers could be attributed to the non-modeled tool deflections, friction, and plastic deformations. Furthermore, a slight geometry-dependent increase in cutting forces and compressive stresses were observed, while a significant reduction in roughness up to 84% and favorable topography conditions were achieved by adjusting wipers and cutting parameters.

## 1. Introduction

Micromachining offers great advantages over conventional machining processes regarding achievable manufacturing precision and surface integrity. In principle, micromachining refers to the scaling of conventional machining processes into the micrometer range [1]. However, since certain influencing factors, such as the material microstructure, are not arbitrarily scalable, size effects arise that significantly distinguish micromachining from conventional machining [2,3]. Micromilling in particular offers the possibility of machining a wide variety of materials and shapes. Even very hard materials, e.g., hardened tool steels, can be machined to a high manufacturing quality. While the achievable surface quality of conventional milling often requires the use of post-processing, micromilling can accomplish the shape production and surface finishing in one process step [4]. Further process steps, such as grinding and polishing, can be avoided in order to shorten the process chain and thus the cycle time of the production. This offers the possibility for tool and mold manufacturing to produce dies and punches with a high degree of quality. However, due to the filigree tools (*d* ≤ 1 mm) and the low values for cutting parameters, high process times occur when machining larger components, which limits the application of the processes. Increasing the feed rate can reduce the process times in conventional machining processes and thus the production costs, however, with a regular tool design, it leads to a negative influence on the surface finish and thus on the production quality [5,6]. Despite the comparatively high material removal rates compared to other processes in microsystems technology, this represents a disadvantage compared to conventional machining.

To overcome this limitation in productivity and achieve a comparable surface finish despite increased tooth feeds, the use of a special tool and cutting-edge geometries has proven successful in the field of conventional machining. Wiper geometries, which have a surface-parallel section of the cutting edge and therefore slightly different engagement conditions, have proven to be particularly successful in the field of turning [7]. This change in tool design achieves comparable surface roughness over an extended range of the selected feed rates. By the application of such advanced tool designs, machining processes can be significantly accelerated. In the field of conventional milling processes, this approach proved to be promising [8]. Therefore, this paper presents a transfer of such a concept to micro end mills. In addition to the simulation-based analyses of the approach, an experimental investigation of modified tools was carried out regarding the achievable surface properties as well as the resulting process forces.

The basic approach of a surface-parallel cut was used in the field of carpentry for planing since the end of the 19th century [9,10]. The specific elements of cutting edge are described as drag cutting edge or parallel land, but mainly referred to as wipers. Typically, wipers are multi-radius design elements of the cutting edge [11]. They can be described by the corner radius, *r*_ε_, the run-out radius, *r*_2_, and the length of the parallel land, *b*_s_, which represents the surface-parallel section of the cutting edge. Furthermore, it can be described as a minor cutting edge, which is parallel in the direction of feed [12].

Such tools or inserts are mainly used in turning operations but are also progressively used on milling tools to allow an increase in feed rate for face milling operations. Kurniawan et al. investigated the use of inserts with wipers in stainless steel turning [13]. In addition to deriving suitable values for cutting parameters and a tool life model for the inserts in focus, they identified negative influences of wiper inserts under unfavorable cutting conditions. For instance, they discovered that at excessive cutting speeds, the edge zone can be negatively affected (white layer) and tensile residual stresses can occur. D’Addona and Raykar analyzed the application in hard turning of tool steel with a hardness of 55 HRC (Hardness, Rockwell C Scale) [14]. They stated the superior surface finish with wipers compared to conventional inserts and identified the feed rate as the most significant parameter for their application. However, it also revealed the great potential for hard machining.

De Souza et al. investigated the application of wiper tools in high-speed face milling of cast iron and analyzed the wear behavior of different wiper geometries [15]. Saleem and Mumtaz analyzed the face milling of the difficult-to-cut material (Inconel 625) with multi-radii wiper inserts and evaluated the tool lifetime and condition of the workpieces [16]. They stated that besides the feed per tooth, a strong influence of the depth of cut on the lifetime of the tool could be identified. Ehsan et al. investigated hybrid wiper inserts for milling heads with a combination of negative as well as positive rake angles [17]. They determined a significantly increased tool life. However, it was shown that the wear was significantly greater with too low a feed rate and that the tools reached the wear criterion more quickly. This again confirms the great influence of the feed rate on the operating behavior of wiper tools. In further investigations, they considered the combination of regular inserts with wiper inserts for further improvement of the resulting surface quality [18]. In addition to the resulting surface roughness, they investigated the wear behavior and the influence on microhardness. The short insight into the application examples for wiper geometries shows the potential of such a design of the cutting edge. Based on this, the transfer to tools for hard micromachining is presented in the following. Within the scope of this investigation, the influence of the tool modification is tested when machining the specimen material, AISI H11. This hot work tool steel (HWS) is one of the most widely used tool steels in the European die casting industry, which due to its thermal shock resistance and high-temperature strength is highly suitable for hot injection molding and die casting processes. The aim of the study is to show whether the wiper geometry approach can be transferred to micromilling cutters and if it offers advantages for machining in mold and die manufacturing. After a brief explanation of the approach as well as the material removal simulation (MRS) used, the experimental setup of the study is presented. Subsequently, the simulative and experimental results are presented and discussed.

### Design Approach for Hard Micromachining of Tool Steels

**Figure 1:**
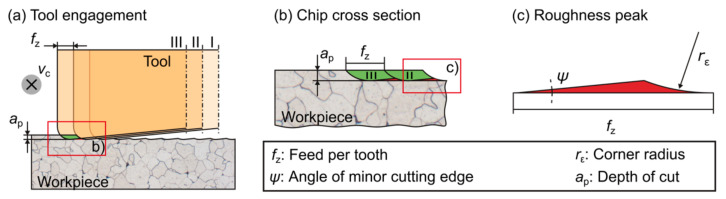
Schematic view of (**a**) a two-dimensional engagement situation during milling, (**b**) two consecutive tooth feeds, (**c**) single milling marks with influencing parameters—as per [20].

The surface roughness of components is an important criterion that clearly determines the later operational behavior of machined functional surfaces [19]. In addition to material-specific effects, the manufactured surface topography of the workpieces and thus the resulting roughness is mainly determined by the engagement conditions [20]. The latter is predominantly defined by the cutting parameters, such as feed per tooth, *f*_z_, depth of cut, *a*_p_, and width of cut, *a*_e_, and the tool design (see Figure 1). This leads to the necessity to adapt the cutting parameter values of the finishing processes to the tolerance specifications of the workpieces in order to meet the design specifications, which can lead to extensive process times.

As depicted in Figure 1, the tool-related design of the cutting edges in combination with the selected cutting parameters defines the chip cross-section (shown in green). Observing successive cuts, it can be seen that a material residue appears on the surface, which results in the process-typical machining grooves or feed marks on the surface. A detailed examination of the resulting roughness peaks illustrates the dependency to process as well as geometric tool characteristics (Figure 1c). In previous research, such considerations of the engagement situation and material residue resulted in several analytical models that estimate the theoretical maximum roughness depth, *Rth*, for different machining processes on the basis of the engagement situation, which was characterized by e.g., tool and process parameters [20,21,22,23].

**Figure 2:**
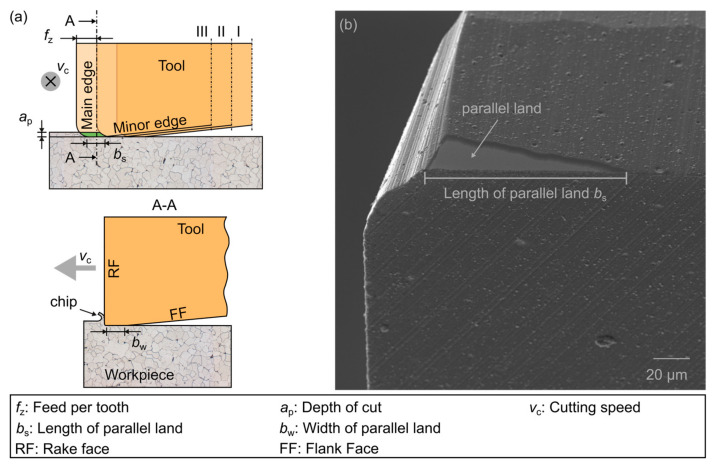
Modified engagement situation of tools with wiper geometry (**a**)—according to [24]; (**b**) preparation result of a tool in the focus of this investigation.

By introducing a surface parallel land of the cutting edge, a change in the engagement conditions can be realized, systematically avoiding the residual material or the machining groove respectively. This changed engagement situation can be seen in Figure 2a. By overlapping the chip cross-sections, and therefore avoiding material residue, the process-related machining groove structure can theoretically be completely avoided. The preparation of the tools also caused a sliding surface, which is comparable to a pre-set wear mark on the flank faces of the tools, as can be seen in Figure 2b.

## 2. Materials and Methods

### 2.1. Material Removal Simulation

The introduced tool concept was investigated using a simulation-based approach in advance to reveal the first dependencies and to restrict the selection of suitable tool modifications for the experimental investigation. For a prediction of the resulting surface topographies with the new tool geometry, a geometrical material removal simulation (MRS) was used [20]. This approach made use of a heightfield as the workpiece representation and a triangle mesh as the tool model, in which the microgeometry of the cutting edges could be included in high resolution. With a time-based discretization of NC milling paths and the rotational speed, discrete substeps of the engagement situations are calculated and the material removal can be simulated via triangle ray intersection tests. The validation of this simulation approach for an application in the field of micromachining was presented in [20].

### 2.2. Simulative Setup

**Figure 3:**
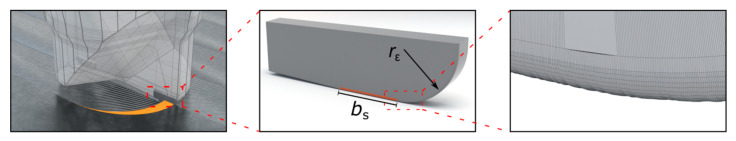
Modeling of the tool design and cutting edge for MRS.

The triangle meshes with different lengths of parallel land, *b*_s_, for the modified tools and a conventional end mill as reference were constructed in a 3D modeling software with the parameters shown in Table 1. The modeling was based on the characterization of the reference tools by means of scanning electron micrographs (Tescan, Mira III XMU, Dortmund, Germany) and a measurement of the cutting edges using a focus variation microscope (Bruker Alicona, Infinite Focus G5, Graz, Austria). Figure 3 depicts an exemplary tool model with the included micro-geometric properties, where *b*_s_ = 60 µm.

The size of the virtual workpiece was set to 1.25 × 1.25 mm with a lateral precision of *d*_D_ = 0.4 µm. For the simulation, an NC path was generated with a width of cut, *a*_e_ = 0.025 mm, and a depth of cut, *a*_p_ = 0.4 mm, in a down milling process. To explore the influence of the modifications on the surface topography and the interaction of the tooth feed, *f*_z_, and the length of parallel land, *b*_s_, process simulations with various values of the feed per tooth in the range of 50 µm ≤ *f*_z_ ≤ 130 µm were conducted for all modeled tools.

### 2.3. Experimental Setup

The experimental investigation was carried out on the machine tool KERN HSPC 2522 (KERN Microtechnik, Eschenlohe, Germany), which is well suited for micromachining due to its high working accuracy of 2.5 μm and a rotational speed range of the tool spindle (VSC 4084 Precise) up to *n* = 50,000 rpm. The acceleration capabilities of the machine tool are specified as *a*_max_ = 2000 mm/s^2^ and a maximum feed rate of *v*_f,max_ = 6000 mm/min are provided. A limitation of external influences, e.g., changes in temperature or vibration, could be achieved by setting up the machine tool in a thermally controlled micro lab at the Institute of Machining Technology on a foundation of polymer concrete.

The investigated tools manufactured by *Seco Tools* were two-fluted end mills (JM103010R005) with a diameter of *d* = 1 mm and a nominal corner radius of *r*_ε_ = 50 μm. The substrate consisting of ultra-fine grain cemented carbide (WC grain size 0.2–0.5 μm) and the applied TiAlN PVD-coating equip these tools for hard machining of tool steels.

**Figure 4:**
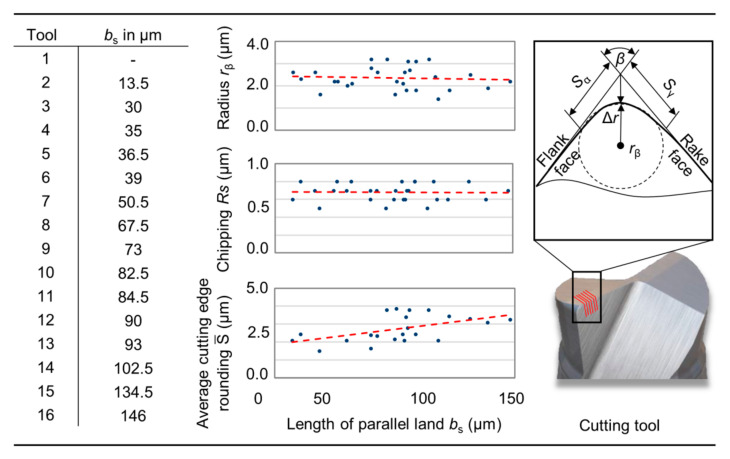
Specification of prepared tools for cutting tests.

Since the microgeometric properties of the cutting edges have a significant influence on the process behavior in micromachining, explicit care was taken to ensure that the condition of the cutting edges was not influenced negatively by the preparation process. After modification of the tools, they were measured and characterized using the fringe projection microscope (LMI Technologies, MikroCAD Plus, Burnaby, BC, Canada). In addition to the determination of the cutting edge radius, *r*_β_, an evaluation was carried out according to Denkena’s form-factor method [25]. The cutting tool geometry was characterized in advance by measuring the prepared length of parallel land, *b*_s_, of each tool using a digital optical microscope (Keyence, VHX-2000, Ōsaka, Japan). The evaluation of the measurement is depicted in Figure 4. It was possible to produce wipers with a length of parallel land between 13.5 µm ≤ *b*_s_ ≤ 146 µm. On average, a mean cutting edge roundness of $\bar{S}$ ≈ 2.92 μm and roughness of the cutting edge of *Rs* ≈ 0.7 µm was achieved, indicating a high-quality condition of the cutting edge.

To evaluate the modifications visually and to determine the initial conditions of the cutting edges, a secondary electron microscope (SEM) (Tescan, Mira III XMU, Dortmund, Germany) was used with a backscattered electron detector (BSE), coplanar energy dispersive X-ray, and electron backscatter diffraction (EDX/EBSD) system. With the aid of an optical focus variation microscope (Bruker Alicona, Infinite Focus G5, Graz, Austria) macrogeometric properties of the tools were digitized to provide sufficient information for the MRS.

When performing the experimental micromilling, the active forces, *F*_a_, and passive forces, *F*_p_, were measured using the MicroDyn 9109AA 3-component dynamometer (Kistler Group, Winterthur, Switzerland), which is specially designed for analyzing micromachining processes due to its high rigidity and the relating high natural frequency of *f*_n_ (x, y, z) = 15 kHz. During the operational testing, the force signals were recorded with a sampling rate of 100 kHz. The process forces were analyzed with a special software tool allowing us to consider, evaluate and quantify the characteristic cutting forces within more than 200 tool engagements. This delivered a sufficient and statistically validated database for the evaluation of the process forces when applying different wipers. A white light microscope µsurf explorer (NanoFocus AG, Oberhausen, Germany) was used to measure the surface topography of the specimens after machining, in order to assess the roughness of the machined workpiece. By evaluating the topography measurements with the aid of the software MountainsMap 7 (Digital Surf, Besançon, France), the arithmetic mean roughness, *Ra*, as well as material ratio curves, could be determined according to DIN EN ISO 4287. For high resolution imaging an Olympus 50× lens with a numerical aperture of *A*_N_ = 0.5 was used. The residual stresses were measured by the use of a diffractometer Advanced D8 (Bruker AXS, Karlsruhe, Germany) equipped with a polycap with a point focus of 2 mm. The applied radiation source was a Cu Kα anode with a photon energy of 8.048 keV operating with a voltage of 40 kV and 40 mA. By the use of the sin^2^ψ method [26] with the Fe (211) reflex, the residual stresses were analyzed. The measurements were conducted for tilt angles of *ψ_t_* = ±0; 7; 18.4; 26.6; 33.2; 39.2; 45 and 60° and rotation angles of *φ* = ±0; 45; 90; 135; 180; 225 and 270°. For the 2*Θ* range, an interval from 80–85° with a step width of *ΔΘ* = 0.1° with an exposure time of 5 s was selected.

### 2.4. Specimen Material for Experimental Investigation

For the experimental investigation specimens of hot work tool steel (HWS) AISI H11 (see Table 2), hardened to approximately 52 ± 1 HRC, were machined with various tools. The high-performance steel offers high toughness and high-temperature strength, low sensitivity to hot cracks, and good thermal conductivity. Therefore, this high-alloyed tool steel is one of the most common materials to be used for dies in hot forming processes.

### 2.5. Design of Experiments

**Figure 5:**
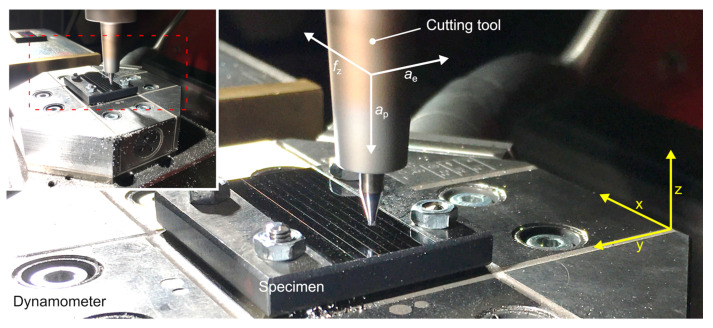
Experimental setup.

For the design of experiments (DoE), a full factorial experimental approach was used to generate a sufficient database with statistical validation within the limits of experimental accuracy. To analyze the achievable manufacturing quality in micromilling with wiper end mills, a variation of the length of the parallel land 13.5 µm ≤ *b_s_* ≤ 146 µm and the feed per tooth 20 µm < *f*_z_ < 120 µm was performed. Due to the feed rate limitation of the machine tool (*v*_f,max_ = 6000 mm/min), the cutting speed, *v_c_*, had to deviate from an adequate value *v*_c,1_ = 120 m/min to *v_c,_*_2_ = 75 m/min for the highest feed per tooth configurations, which must be taken into account when interpreting the results. The remaining set of cutting parameters was kept constant with a depth of cut *a*_p_ = 0.025 mm and a width of cut *a*_e_ = 0.4 mm. Based on this parameter set, *n* = 102 (17 tools × 6 feed per tooth configurations) experiments were conducted. The respective parameter set and the related experimental setup are shown in Table 3 and Figure 5.

## 3. Results and Discussion

For an adequate illustration, first, the results of the MRS are processed in the design and analysis of computer experiments (DACE) models to show a holistic influence of the wiper geometry on the resulting surface topography. Subsequently, the experimental results of the obtained surface characteristics and process forces, as well as SEM images of the tools used, are presented and discussed.

### 3.1. Material Removal Simulation

**Figure 6:**
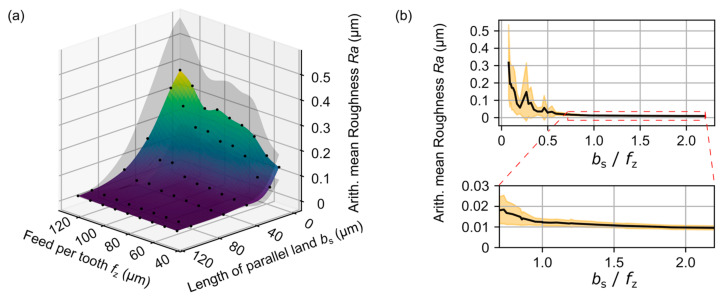
(**a**) depicts the arithmetic mean roughness, *Ra*, of the surfaces, generated with the MRS using different tool models with various wiper lengths and the reference tool. The factor *b*_s_/*f*_z_ and the corresponding *Ra* are shown in (**b**).

In this section, the results from the MRS are presented. A positive impact of the modeled wiper tools on the surface roughness and a correlation between the feed per tooth, *f*_z_, and the length of parallel land, *b*_s_, could be determined. The occurred arithmetic mean roughness, *Ra*, for the simulated surfaces is presented in Figure 6, showing a significant positive impact of the new tool shape on the surface roughness.

In comparison to the reference tool, the resulting surface roughness could be reduced with all modified tools over the entire simulated range of the feed per tooth, *f*_z_, configurations. Furthermore, a clear correlation between the feed per tooth, *f*_z_, and the length of parallel land, *b*_s_, could be detected. If the length of the parallel land, *b*_s_, is higher than the feed per tooth, *f*_z_, a near-constant low level of the surface roughness is achieved. After the feed per tooth exceeds the length of the parallel land, the arithmetic mean roughness value, *Ra*, increases, but stays beneath the values achieved with the reference tool. This correlation can be described by the quotient *b*_s_/*f*_z_ which is applied in Figure 6. It shows low resulting roughness values of *b*_s_/*f*_z_ ≥ 0.5. Considering the flattening of the curve above *b*_s_/*f*_z_ = 1, an upper limit can be set by 1.5 after which the effect on the surface roughness can be neglected. The optimal length of the parallel land can be determined by 0.5 *f*_z_ ≤ *b*_s_ ≤ 1.5 *f*_z_.

**Figure 7:**
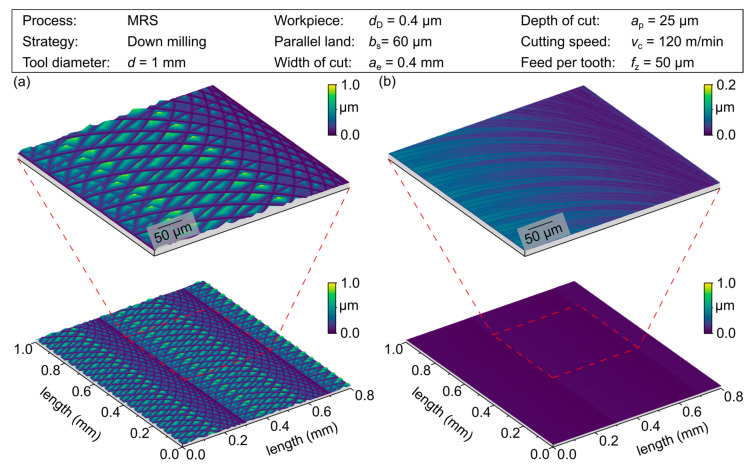
Surface topography simulated by material removal simulation (MRS) with (**a**) reference tool and (**b**) wiper tool with *b*_s_ = 0.06 mm for *f*_z_ = 0.05 mm.

The influence of the tool modification is further illustrated by the topographies of the simulated surfaces, shown in Figure 7. The surfaces of a reference tool and a modified tool with a length of *b*_s_ = 60 µm at a feed per tooth of *f*_z_ = 50 µm can be seen. While the surface generated with the reference tool shows the typical milling grooves, the changed tool geometry could improve the surface topography, due to the surface parallel engagement of the cutting edge. This leads to a significant decrease in the influence of the macrogeometric properties of the tool and thus an increase in the influence of its microgeometric characteristics. The topography of the surface is thus largely determined by the condition of the cutting edge, e.g., the chipping of the cutting edge, *R*s.

### 3.2. Experimental Investigations

In the following, the discussion of the experimental results is divided into individual sections. First, the surface topographies and determined roughness values (3.2.1) are presented and discussed in relation to the length of the influencing factor of parallel land, *b_s_*, and feed per tooth, *f*_z_. Thereafter, the cutting forces and the residual stresses are analyzed and explanatory approaches are derived (3.1.2).

#### 3.2.1. Surface Topography and Roughness Characteristics

**Figure 8:**
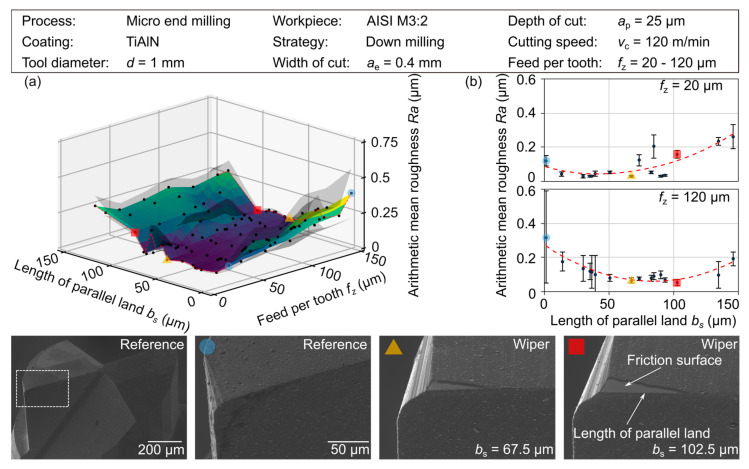
(**a**) Arithmetic mean roughness, *Ra*, for different values of the length of parallel land, *b*_s_, and the varied feed per tooth, *f*_z_; (**b**) Influence of increasing length of parallel land, *b*_s_, on resulting roughness at *f*_z_ = 20 µm and 120 µm.

In the following section, the influence of different sizes of wiper geometries on the resulting surface topography when machining the HWS AISI H11 is discussed regarding the relationship between the length of parallel land and the feed per tooth *(b_s_/f*_z_*)*. The specimens were face milled to ensure a defined depth of cut for the cutting tests and the dimension of 32 × 32 mm allows the arrangement of six tests with a tool path length of l = 75 mm (5 × 15 mm) each. Figure 8. depicts the determined arithmetic mean roughness, *Ra*, is an interpolated DACE model, which is qualitatively supplemented by SEM images of the prepared cutting edges. As can be seen in the results depicted in Figure 8, it was possible to confirm the positive influence of the wiper geometry on the resulting surface roughness over the entire observed set of parameter values within the experimental investigation. Furthermore, the obtained roughness values for the experimental investigations are shown in Table 4.

Initially, the analysis of the wiper geometry is concentrated on a low feed per tooth of *f*_z_ = 20 µm. For a conventional tool geometry, the achievable arithmetic mean roughness was *Ra*,_ref_ = 0.122 µm. However, even a rather small wiper with a length of parallel land of *b_s_* = 13.5 µm had a significant effect on the surface topography. In the experimental test a roughness *Ra*,_13.5_ = 0.043 µm could be determined, which represents a reduction of the arithmetic mean roughness of 65%. With an increasing length of the parallel land, *b_s_*, a further reduction of the considered roughness value, *Ra*, can be achieved. For the lengths *b_s_* = 67.5 µm and *b_s_* = 90 µm, the value of *Ra* = 0.029 nm results in a reduction of 76%.

However, some tools with large wiper geometries also showed a significant negative effect on the surface roughness values at lower feed per tooth rates. When exceeding a certain length of parallel land (*b_s_ >* 80 µm), the surface roughness increases, which indicates the significance of further effects in the application of wiper geometries. Using a length of parallel land of *b_s_* = 102.5 µm led to roughness values above the reference. This was explained by the increase in the contact area caused by the wiper geometry of the tool, which interacts with the elastically recovered workpiece material. As the surface area increase, a higher portion of friction in the process can be assumed, resulting in surface defects and an increase of surface roughness. While the experimental results regarding roughness characteristics are in high agreement with the MRS results for a large range of experiments, this trend shows a deviation for very high values of the length of parallel land, *b_s_*. This was justified by the model characteristics of the MRS, which only considers the engagement situation and tool geometry, but neglects material-specific effects such as friction or ploughing.

**Figure 9:**
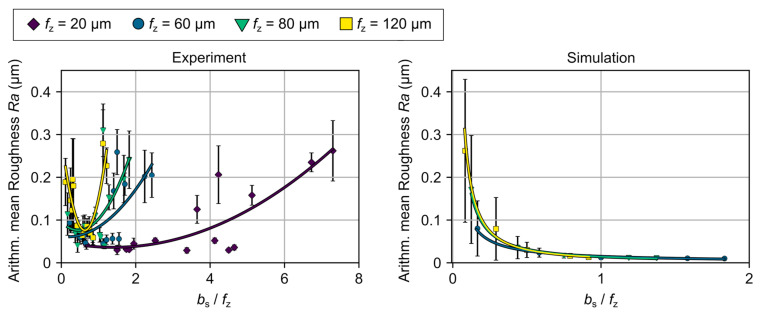
Comparison of the simulated and experimentally determined ratio of the parallel land length, *b*_s_, and the feed per tooth, *f*_z_.

While at large wipers, a slightly negative development of the surface roughness can be determined for low values of the feed per tooth, a positive development can be observed for high values. This suggests that the tools with wiper geometries have an optimal application range, which is depending on its length of parallel land and is determined by both a minimum and a maximum sensible feed per tooth. While friction effects have an impact if the feed rate is selected too low, the engagement situation changes if the feed rate is selected too high. Here, the influence of the macrogeometric properties increases again resulting in a deterioration of the surface quality. The exemplarily illustrated trend lines of *Ra* over the length of the parallel land, *b_s_*, (Figure 8), depict a shift of the determining process window to higher values when rising the length of parallel land, *b_s_*, to a comparable value of the feed per tooth. Here, the previously mentioned quotient *b*_s_/*f*_z_ becomes apparent once again. If for example a conventional cutting tool is used with a feed per tooth of *f*_z_ = 120 µm, values for the arithmetic mean roughness up to *Ra*,_Ref_ = 0.375 µm arise as expected. In contrast, for a small and a large wiper with *b_s_* = 13.5 and 102.5 µm, roughness values of *Ra*,_13.5_ = 0.189 and *Ra*,_102.5_ = 0.0588 µm were determined, representing a reduction of 49.6% and 84.3%, respectively. While the tool with the small wiper is used with a quotient of *b*_s_/*f*_z_ = 0.11 and thus runs at a very high feed rate compared to the length of the parallel land, *b*_s_, the tool with the bigger wiper is used with a quotient *b*_s_/*f*_z_ = 1.0. The tool is therefore working closer to its optimum application. In Figure 9, a high agreement between the simulated and the experimentally determined ratio of the length of the parallel length, *b*_s_, and the feed per tooth, *f*_z_, can be observed. This is limited by a factor *b*_s_/*f*_z_ < 1, which is due to the observed effects especially for larger wiper geometries, and the limitation of the MRS, which does not consider tribological and material-specific effects. Furthermore, it is particularly noticeable that the standard deviation of a respective surface produced by tools with wiper geometry using a suitable process window is remarkably low compared to the conventional tool geometry, see Table 4. The considerable reduction in the exemplarily described feeds per tooth of *f*_z_ = 20 and 120 µm from s_ref,20_ = 37 nm and s_ref,120_ = 173 nm to extremely low values of s_bs36,20_ = 3 nm and s_bs93,120_ = 12 nm reinforce the potential additionally by a high process capability.

Considering the setting of the experiments, the respective tools with similar parameters and values for the length of the parallel land show differences in surface finish, which may be due to uneven embossing of the geometry between the respective cutting edges caused by inaccuracies in the preparation process. Nevertheless, in large areas for the feed per tooth the wiper geometry in hard machining shows a substantial potential for the considered tool geometry and workpiece material.

**Figure 10:**
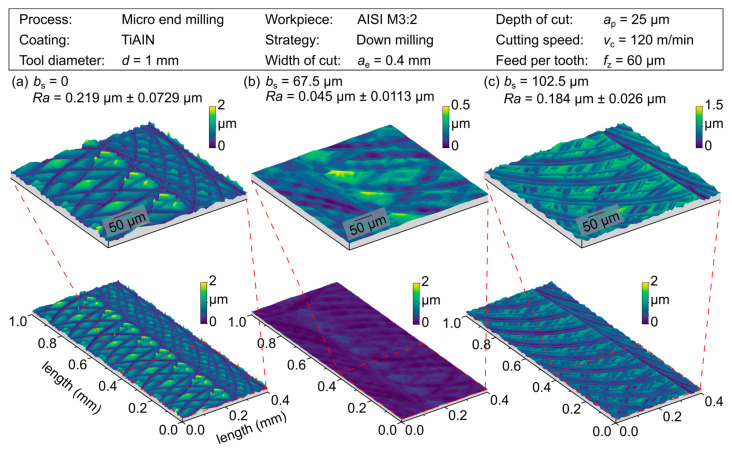
Surface topography for the parallel land *b_s_* = 0, 67.5, 102.5 µm.

**Figure 11:**
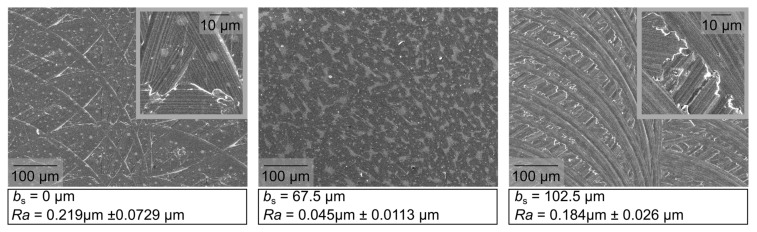
SEM measures of the surface finish for the parallel land *b_s_* = 67.5 µm and 102.5 µm.

To evaluate the measured differences in surface roughness, a visual evaluation of the surface topography was carried out, see Figure 10. Compared to the micromilling results using the conventional tool geometry, wipers with a length of the parallel land of *b_s_* = 67.5 and 102.5 µm have been exemplarily analyzed at a feed per tooth of *f*_z_ = 60 µm. see Figure 10 (a) shows the surface machined with the conventional tool shape resulting in the expected milling grooves. In addition, a dominant proportion of roughness peaks can be observed, which increases the surface roughness characteristics compared to the simulated surface in Figure 7. The resulting topography can be explained by different cutting sequences, which contain a certain degree of overlap [20]. Due to a disadvantageous combination of different tool engagements, resulting topography peaks determine the resulting surface. In contrast, Figure 10 (b) shows a smooth “wiped” surface topography with roughness values comparable to a polished surface, which was machined at a quotient of *b*_s_/*f*_z_ = 1.13. Related to the theoretical assumption that wipers produce an almost perfectly flat surface, there are non-ideal boundary conditions, e.g., manufacturing-related deviations as well as static and dynamic deflections of the tools due to the mechanical process load, etc., that cause the resulting surface topography. The resulting topography is composed of low peaks accompanied by slight grooves that dominate the micro roughness. An unfavorable ratio of *b_s_/f*_z_ = 1.71 can be observed in Figure 10 (c) depicting wide milling grooves overlapping an undefined grooved surface. The path of the grooves has a change in its orientation and thus shows a variation in cutting conditions, compared to the surfaces shown before. This was justified by high cutting forces, imposing an inclination of the tool. In addition, a fringed rim and ground of the cutting grooves show a portion of plowing, especially in the SEM images (Figure 11).

This supports the assumption of high portion plastically deformed volume and a higher degree of friction, which increases with the length of parallel land based on an enlarged contact area behind the cutting edge. Due to the complex engagement of the tool geometry in the workpiece material, and influence of further aspects, such as the cutting force and thus the tool wear or the induced stress state, can be assumed.

**Figure 12:**
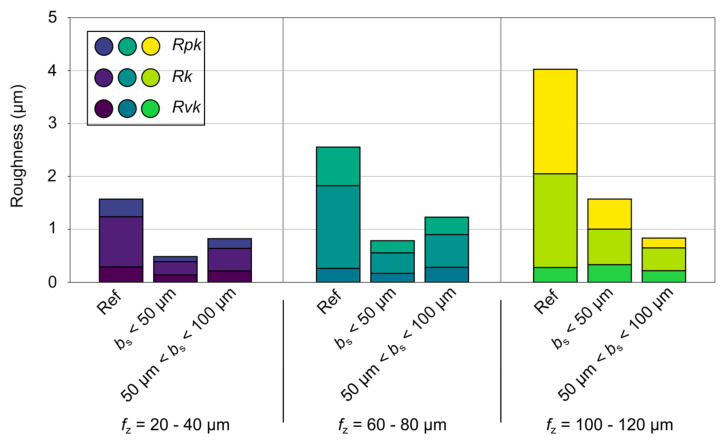
Surface characteristics with the parameters core roughness depth (*Rk*), reduced peak height (*Rpk*), and reduced valley depth (*Rvk*).

Figure 12 shows the average surface characteristics of grouped wiper geometries described in core roughness depth (*Rk*), reduced peak height (*Rpk*), and reduced valley depth (*Rvk*), also known as bearing area ratio. In this context, the considered lengths of the parallel land were summarised as *b*_s_ < 50 µm and 50 < *b*_s_ < 100 µm for the varied feeds per tooth *f*_z_ = 20–40 µm, *f*_z_ = 60–80 µm, and *f*_z_ = 100–120 µm. With respect to the reference geometry, the feed per tooth 20 µm < *f*_z_ < 40 µm leads to high values of core roughness, *Rk*, accompanied by low values of reduced peak height and reduced valley depth with an average ratio of *Rvk* to *Rpk* of approximately 1.14. As the feed per tooth increases into the range of 100–120 µm, the values of *Rk* and *Rpk* increase sharply, while *Rvk* remains low, resulting in a ratio of *Rvk*/*Rpk* = 7.02, which means a peak-dominated surface characteristic. In comparison, the small wipers show a strong influence on the resulting surface finish. A significant reduction of the parameter values can be observed over the entire feed per tooth range investigated. In particular, the defined process window close to the length of the parallel land, *b*_s_, leads to favorable ratios of *Rvk*/*Rpk* of approx. 0.69, which increases to 1.7 by exceeding this area at a higher feed per tooth. Due to the low influence of the feed per tooth on the surface quality, the large wipers show the highest process capability with values of 0.86 for *Rvk*/*Rpk*, which decreases to 0.84 with a feed per tooth *f*_z_ = 100–120 µm. In summary, by using wiping tools, a favorable surface characteristic that has a smooth plateau surface with very small valleys can be achieved.

#### 3.2.2. Cutting Force and Residual Stress State

**Figure 13:**
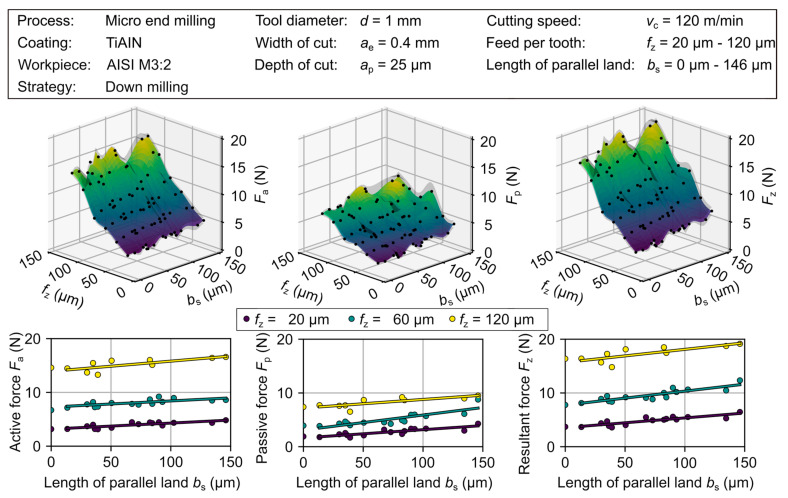
Resulting cutting forces subdivided according to passive and active force components.

The following section discusses the influence of a variation of the tool geometry as well as the feed per tooth on the resulting cutting force. Figure 13 depicts the determined active, *F*_a_, passive, *F*_p_, and the resulting cutting forces, *F*_z_, of the experimental investigation. It should be mentioned that due to the limited machine tool capabilities (*v*_f,max_ = 6000 mm/min), the realisation of a feed per tooth *f*_z_ = 100, 120 µm required a reduction of the cutting speed from *v*_c,1_ = 120 m/min to *v*_c,1_ = 75 m/min. For the reference tool, an increase of all force components over the observed range of the feed per tooth *f*_z_ = 20–120 µm was determined. Over the analyzed feed range the active force increased from *F*_a_ = 3.2 N to *F*_a_ = 14.6 N, the passive force from *F*_p_ = 1.9 N to *F*_p_ = 7.4 N, and the resulting force from *F*_z_ = 3.7 N to 16.4 N. Due to the adapted cutting speed an additional increase of the forces at *f*_z_ = 100, 120 µm occurred.

When analyzing the wiper tools, it is important to keep in mind that, despite efforts not to influence the shape of the cutting edge, some effect of the preparation is to be expected. A divergent shape of the cutting edge may change the engagement situation. Furthermore, due to the introduced changes in the tool geometry, a higher degree of friction and plastic material deformation is to be expected. As a result, a slight increase in the cutting force components can be observed for higher values of the length of parallel land, *b*_s_. Over the observed range of the feed per tooth of *f*_z_ = 20–120 µm, an increase of the parallel land, *b*_s_, shows an approximately linear correlation of the resulting cutting forces, *F*_z_, see Figure 13. The following presentation of the results is therefore done in a partial summary of the length of parallel land, *b*_s_, to small wiper geometries 0 µm < *b*_s_ < 50 µm and large wiper geometries 50 µm < *b*_s_ < 146 µm.

In the following, the change in cutting force is discussed in comparison to the results of the reference tool. Small wiper geometries show an increase of +8.4% to *F*_a_ = 3.44 ± 0.33 N for the active force at a small feed per tooth of *f*_z_ = 20 µm, while the passive component increases by +10.3% *F*_p_ = 2.10 ± 0.32 N, resulting in minor changes in the resulting cutting force of 9% up to *F*_z_ = 4.04 ± 0.44 N. When increasing the feed per tooth to *f_z_* = 120 µm, the resulting cutting force related to the reference cutting tool showed no difference for the active component and an increase of +3.4% to *F*_p_ = 7.60 ± 0.77 N for the passive component. It was therefore possible to determine that the tool modifications had only a minor influence on the process forces.

Taking into account the large wiper geometries, significantly stronger influences of the modified tools were detected. The active force component shifted about 37% to *F*_a_ = 4.34 ± 0.27 N and an increase in the passive force of +66% to *F*_p_ = 3.16 ± 0.53 N was observed for a feed per tooth of *f*_z_ = 20 µm. This led to a change in the resulting cutting force of up to +45% to *F*_z_ = 5.39 ± 0.46 N. Again, taking into account the feed per tooth up to *f*_z_ = 120 µm, the resulting cutting force relative to the conventional cutting tool shows an increase of +10% to *F*_a_ = 16.0 ± 0.66 N, an increase of +23% to *F*_p_ = 9.09 ± 0.36 N and an overall difference of +12.8% to *F*_z_ = 18.46 ± 0.72 N for the resulting cutting force.

In summary, a feed per tooth of *f*_z_ = 20 µm can be considered not critical for most wiper geometries, as the active force increased slightly. Bigger wiper geometries showed the worst results with this parameter set. However, the small wiper geometries have favorable force ratios over a wide range of feed per tooth and partially showed only minor negative influence on the resulting cutting force. Thus, a process window can be considered that does not show any limitations due to the tool load for the parameters investigated. This confirms the potential of wiper modification since for an advantageous process design the negative influences are reasonably low despite the significantly adjusted engagement situation. In contrast, large wipers showed unfavorable force ratios, especially at low feed per tooth configurations, with an increased tool load and presumably reduced tool life, even when the absolute difference in values is close to 1.6 N. This negative effect decreases as the feed per tooth increases, which can be considered as a process window that depends on a trade-off between the cutting forces and the associated productivity and surface topography. However, such approaches can be useful for roughing processes in which mainly a high material removal rate is to be achieved and additional process objectives are neglected. In this context, an investigation of tool wear would be very valuable, which could provide information on the productivity factor. Furthermore, the differences in cutting forces may have an impact on the resulting surface quality in terms of tool wear and surface quality, which is to be analyzed in further investigations.

**Figure 14:**
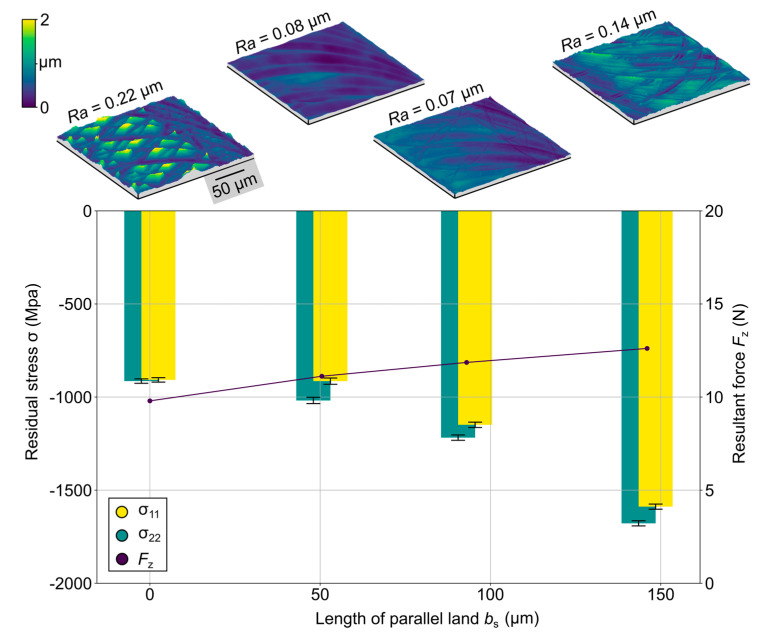
Residual stresses in the subsurface zone of the steel substrate.

With regard to the measured cutting forces, the resulting residual stress states for certain lengths of the parallel land, *b*_s_, at a constant feed per tooth of *f*_z_ = 80 µm were investigated to map advantageous and disadvantageous ratios of *b*_s_ to *f*_z_ (Figure 14). For the residual stress tests, the specimens were surface ground to produce a typical residual stress condition as reference.

With an extension of the length of the parallel land, *b*_s_, an increase of the compressive residual stress state can be observed, which corresponds to the resulting cutting forces dominated by the passive component. This was justified by the increased contact area between the tool and the workpiece as well as stronger plastic deformation of the workpiece material. Consequently, an influence on the residual stress state can be assumed. These results underline the high potential of a wiper geometry for both the surface topography as well as the boundary zone, which promotes further investigations of the applications of wiper geometries in micromachining.

## 4. Conclusions

In this study, the potential of wiper geometries for micromilling of hardened HWS (AISI H11) was analyzed by simulation-based and experimental investigations. The process performance of modified micro end mills with varying lengths of the parallel land, *b*_s_, was evaluated in a parameter study in which the feed per tooth, *f*_z_, was altered in the range 20 µm < *f*_z_ < 120 µm. Within the scope of the analysis, the simulated and manufactured surface topographies were examined and the impact of the tool modification on the process behavior was determined on the basis of the occurring cutting forces as well as the resulting residual stress state of the specimen’s subsurface zone. Based on the results obtained in this work, it can be concluded that:

- The simulation-assisted development of micro end mills with wiper geometries demonstrated a high potential based on the visualization of the achievable surface finish.
- Compared to the conventionally designed cutting tools, the wiper tools showed a significant improvement in the achievable surface topography for determined ratios of the parallel land length and the feed per tooth *b*_s_/*f*_z_. This included a reduction of the arithmetic mean roughness up to 75% (*R*a = 29 nm) and 84% (*R*a 59 nm) for the values of the feed per tooth *f*_z_ = 20 and 120 µm with a remarkably low standard deviation.
- The measured cutting forces showed a minor impact of the modification, e.g., the length of parallel land, *b*_s_.
- Higher values for the compressive residual stress could be observed with increased wiper geometry.
- In addition, wiper geometries enable higher material removal rates and thus an increase in productivity at enhanced surface quality.

Due to the potential of wiper proven in micromilling, it seems well worth encouraging further investigation:

- In order to obtain essential information about relevant influencing factors, a significance analysis based on a parameter study for relevant cutting parameters, especially for the cutting speed, is of interest.
- The consideration of the tool’s lifetime and the specific wear mechanism and different materials can provide evidence of the degree of permanence of the positive effects observed.
- The demonstrated influence on the stress state shows a high potential for the topic of “surface integrity”, which could be further investigated in terms of induced stress state and micro hardness in combination with favorable surface topographies.
- Preparation of the cutting edge of tools with wiper geometry by wet abrasive jet machining to achieve a higher resistance to mechanical load.
- Occurrence or elimination of dynamic effects due to additional friction and damping caused by the increased contact surface behind the cutting edge has not yet been investigated and could be interesting for both micro and macro machining.

## Figures and Tables

**Table 1:** Parameters of the modelled tools with various wiper lengths.

Properties	Value
Tool diameter	*d* = 1 mm
Corner radius	*r*_ε_ = 50 µm
Cutting edges	*z*_n_ = 2
Rake angle	*γ* = 0°
Clearance angle	*α* = 10°
Mean cutting edge roundness	$\bar{S}$ = 2.3 μm
Roughness of cutting edge	*R*s = 0.7 µm
Parallel land	*b*_s_ = 0, 10, 35, 60, 95, 110 µm

**Table 2:** Composition of alloying element in AISI H11 (values specified in wt%).

C	Si	Mn	Cr	Mo	V
0.38	1.00	0.40	5.30	1.20	0.40

**Table 3:** Experimental Design (full factorial).

Length of Parallel Land (*b_s_*)	Feed per Tooth (*f*_z_)	Cutting Speed (*v*_c_)
13.5–146 µm	20–80 µm	120 m/min
	100–120 µm	75 ^1^ m/min

^1^ Further increase of the feed per tooth, *f*_z_, required a reduction of the cutting speed, *v_c_*, due to limitations of the machine tool capabilities.

**Table 4:** Arithmetic mean roughness, *Ra*, and the corresponding standard deviation for the experimental tests performed.

	*Ra* (nm)					
*b*_s_ in µm	*f*_z_ = 20 µm	*f*_z_ = 40 µm	*f*_z_ = 60 µm	*f*_z_ = 80 µm	*f*_z_ = 100 µm	*f*_z_ = 120 µm
Reference	117 ± 37	239 ± 98	219 ± 73	335 ± 129	324 ± 139	375 ± 173
13.5	43 ± 11	71 ± 19	93 ± 19	114 ± 50	174 ± 57	189 ± 55
30.0	29 ± 11	49 ± 17	59 ± 16	70 ± 23	135 ± 48	146 ± 76
35.0	32 ± 5	50 ± 12	77 ± 32	41 ± 17	118 ± 19	123 ± 48
36.5	31 ± 3	45 ± 12	64 ± 17	75 ± 22	116 ± 64	195 ± 96
39.0	45 ± 13	33 ± 5	47 ± 10	75 ± 15	101 ± 24	180 ± 110
50.5	52 ± 6	43 ± 8	60 ± 19	86 ± 26	80 ± 28	87 ± 19
67.5	29 ± 6	39 ± 5	45 ± 11	57 ± 9	63 ± 18	64 ± 15
73.0	125 ± 33	55 ± 12	54 ± 11	111 ± 19	76 ± 16	71 ± 11
82.5	52 ± 6	41 ± 7	57 ± 13	65 ± 17	78 ± 22	86 ± 17
84.5	206 ± 68	29 ± 6	168 ± 42	60 ± 11	90 ± 24	82 ± 22
90.0	29 ± 5	40 ± 3	259 ± 53	310 ± 62	99 ± 20	84 ± 24
93.0	36 ± 6	40 ± 9	56 ± 15	40 ± 7	67 ± 18	69 ± 12
102.5	158 ± 23	166 ± 41	184 ± 26	153 ± 29	51 ± 13	59 ± 16
134.5	235 ± 22	169 ± 30	202 ± 61	196 ± 56	96 ± 17	279 ± 79
146.0	262 ± 71	184 ± 44	205 ± 52	241 ± 67	192 ± 58	227 ± 42

## Data Availability

The data presented in this study are available on request from the corresponding author. The data is not publicly available due to the required approval of the cooperating party in the transregional collaborative research center TR73.

## Nomenclature

*A*_N_	Numerical aperture	*Rpk*	Reduced peak height
*a*_e_	Width of cut	*Rth*	Theoretical max. roughness depth
*a*_max_	Acceleration	*Rs*	Roughness of cutting edge
*a*_p_	Depth of cut	*Rvk*	Reduced valley depth
*b*_s_	Length of parallel land	*r*_2_	Run-out radius
*b*_w_	Width of parallel land	*r*_β_	Radius
DoE	Design of Experiments	*r*_ε_	Corner radius
*d*	Diameter	$\bar{S}$	Mean cutting edge roundness
*d*_D_	Lateral precision of highfield	SEM	Secondary electron microscope
*F*_a_	Active force	*s*	Standard deviation
FF	Flank face	*S_α_*	Cutting edge segment on flank face
*F*_p_	Passive force	*S_γ_*	Cutting edge segment on rake face
*F*_z_	Resulting force	*v*_c_	Cutting speed
*f*_n_	Natural frequency	*v*_f,max_	Maximum feed rate
*f*_z_	Feed per tooth	*z*_n_	Cutting edges
HRC	Hardness Rockwell C Scale	*α*	Clearance angle
HWS	Hot work tool steel	*β*	Wedge angle
*l*	Tool path length	*γ*	Rake angle
MRS	Material removal simulation	*Δr*	Radius delta
*n*	Rotational speed range	*σ*	Residual stress
*Ra*	Arithmetic mean roughness	*φ*	Rotation angle
RF	Rake face	*ψ*	Angle of minor cutting edge
*Rk*	Core roughness depth	*ψ*_t_	Tilt angle
